# Supplementary material for: Fiber-specific structural properties relate to reading skills in children and adolescents
Source: eLife. 2022 Dec 28;11:e82088. doi: 10.7554/eLife.82088 (PMC9815823; doi:10.7554/eLife.82088)
Supplement: Supplementary file 1. — Group comparison columns list significant t-statistics. *p<0.05 for the ANOVA between all sites. Post hoc t-tests were only run if the between-sites ANOVA was significant. Only significant t-statistics (p<0.05) are shown in the table. A positive t-statistic denotes Site 1 > Site 2. EHI, Edinburgh Handedness Inventory; SES, socioeconomic status; ICV, intracranial volume; TOWRE, Tests of Word Reading Efficiency composite score, age-normalized; WISC VSI, Wechsler Intelligence Scale for Children visuospatial index, age-normalized; WISC VCI, Wechsler Intelligence Scale for Children verbal comprehension index, age-normalized; gFD, globally averaged fiber density; gFC, globally averaged fiber cross-section. [file elife-82088-supp1.pdf]

Supplementary Material

Metric	F-statistic	CBIC vs. CUNY t-stat	CUNY vs. RU t-stat	RU vs. SI t-stat	CBIC vs. RU t-stat	CUNY vs. SI t-stat	CBIC vs. SI t-stat
Age	2.30	-	-	-	-	-	-
EHI	0.12	-	-	-	-	-	-
SES	10.67*	-	-	2.81	3.53	3.51	3.72
ICV	19.47*	-2.24	5.01	-	6.50	4.05	3.23
WISC VCI	8.06*	-	-	-	4.72	-	-
WISC VSI	9.31*	-	-	-	5.17	-	-
TOWRE	2.30	-	-	-	-	-	-
gFD	152.8*	-9.63	-	1.30	-1.97	1.04	4.30
gFC	4.04*	-	-	2.11	2.09	2.79	2.84
Motion	35.83*	-	-	-5.17	-8.11	-4.47	-7.61
Neighbor Corr.	11.59*	-5.82	5.24	-	-	2.99	-

Supplementary File 1. ANOVA results for site-wise comparisons between phenotypic and neuroimaging metrics. Group comparison columns list significant t-statistics. * denotes $p < 0.05$ for the ANOVA between all sites. *Post-hoc t*-tests were only run if the between-sites ANOVA was significant. Only significant t-statistics ($p < 0.05$) are shown in the table. A positive t-statistic denotes Site 1 > Site 2. *Abbreviations*: EHI - Edinburgh Handedness Inventory; SES - socioeconomic status; ICV - intracranial volume; TOWRE - Tests of Word Reading Efficiency composite score, age-normalized; WISC VSI - Wechsler Intelligence Scale for Children visuospatial index, age-normalized; WISC VCI - Wechsler Intelligence Scale for Children verbal comprehension index, age-normalized; gFD - globally-averaged fiber density; gFC - globally-averaged fiber cross-section.
